# Atypical chronic lymphocytic leukemia with biallelic 13Q deletion

**DOI:** 10.1016/j.htct.2026.106457

**Published:** 2026-04-15

**Authors:** Verónica Roldán Galiacho, Leticia Altuna Tellechea, Marta Alonso Varela, Ignacio Diez de Lezcano Manrique deLara

**Affiliations:** a Department of Hematology, Hospital Universitario Cruces, Barakaldo Bizkaia, 48903, Spain; b Department of Clinical Analysis, Hospital Universitario Cruces, Barakaldo Bizkaia, 48903, Spain; c Department of Immunology, Hospital Universitario Cruces, Barakaldo Bizkaia, 48903, Spain; d Department of Pathology, Hospital Universitario Cruces, Barakaldo Bizkaia, 48903, Spain

A 73-year-old woman presented with an ulcer of the right foot. Physical examination revealed adenopathies. On admission, a blood test showed hemoglobin 78 g/L, platelets 185 × 10^9^/L, leukocytes 451 × 10^9^/L (lymphocytes 447.2 × 10^9^/L), and lactate dehydrogenase 739 U/L (normal range: 125–264 U/L); without alterations of the other hemolytic parameters. A peripheral blood smear displayed atypical lymphocytes some small with ‘turtle-like’ chromatin and coarse cytoplasm; but others were of medium-large size with occasional vacuoles, some of which had lobated or flower-shaped nuclei (Figure 1A-C). Flow cytometry confirmed the clonality: positive for CD19, CD20^low^, CD43, CD79^low^, CD22 and CD200 with kappa chain restriction and negative for CD5, CD10 and CD38. Fluorescence in situ hybridization (FISH) demonstrated a biallelic 13q deletion (Figure 1D). The *IGVH* and *TP53* genes were unmutated. A Positron Emission Tomography/Computed Tomography study demonstrated supra- and infradiaphragmatic adenopathies (standardized uptake value **[**SUV]_max_​ 1.5–5.0) and a lytic lesion in the left ilium (SUV_max_​ 4.3). Histopathological analysis of the bone biopsy revealed an infiltration of lymphocytes positive for CD19, CD23, and ZAP70, and negative for Cyclin D1, CD10, CD38, and BCL6. These findings are diagnostic of atypical chronic lymphocytic leukemia (aCLL); high-grade transformation to large cell lymphoma was excluded.

**Figure 1:**
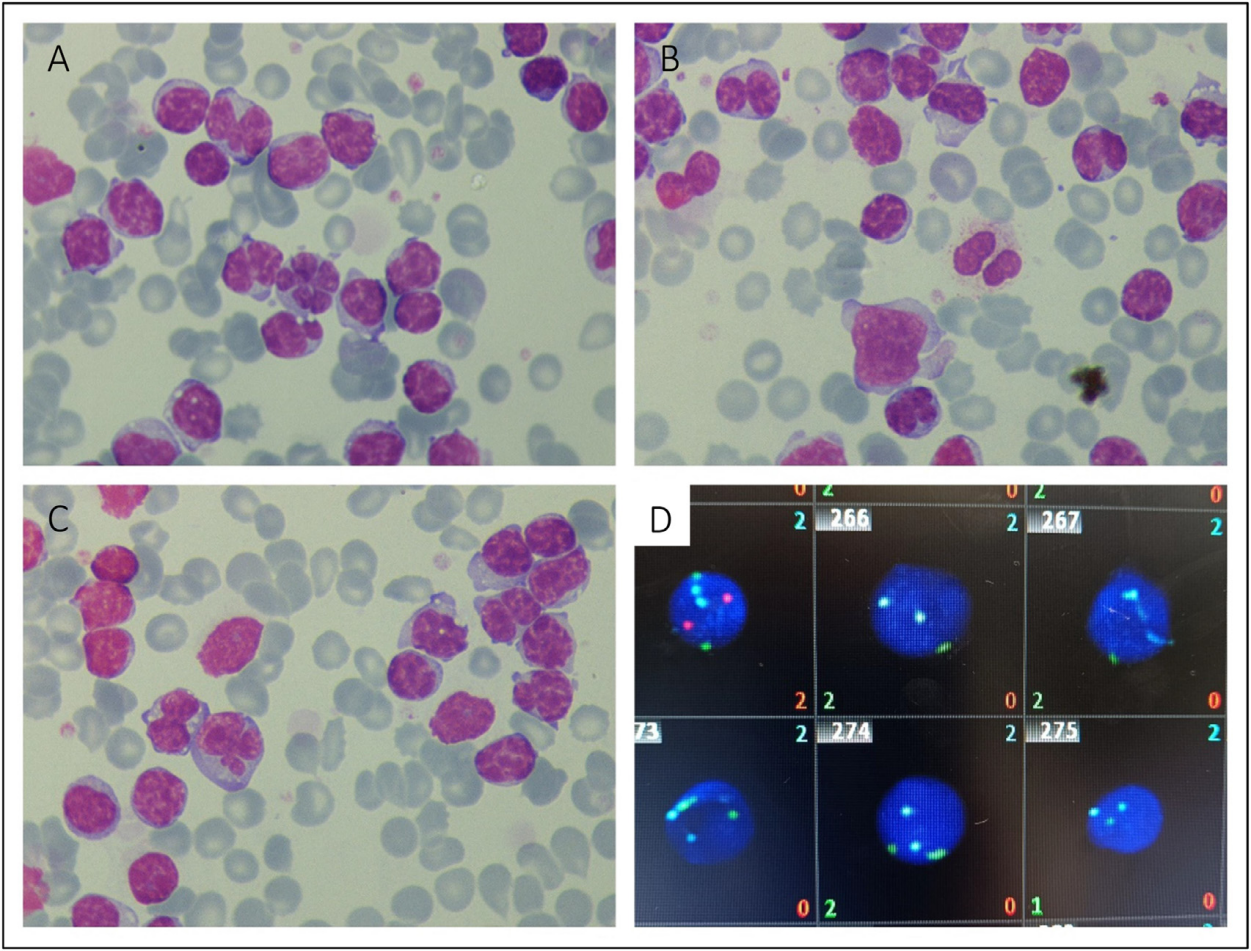
A-C. Peripheral blood smear, May-Grunwald Giemsa x100. D. FISH, CLL Probe Kit: XL DLEU/LAM/12cen showing 5 abnormal cells with two green and two blue signals; and one with a normal pattern: 2G2R2B-upper left-.

aCLL may be defined by morphological (large lymphocytes, nuclear irregularities with or without nucleoli) or phenotypical characteristics (lack of expression of antigens). This is most frequently described in patients with markers associated with poor prognosis like trisomy 12 or CD38 expression [1,2]. Monoallelic 13q deletion is the most common cytogenetic abnormality in CLL; biallelic deletions are rare [3].

We report this case due to its atypia, which raised the possibility of a leukemic phase of a large cell lymphoma, accompanied by elevated LDH levels and adenopathies. This case highlights the importance of an integrated diagnosis, including a pathological examination of suspected lesions to rule out large cell lymphoma.

## Author contributions

Verónica Roldán Galiacho performed the morphological examination of peripheral blood, FISH analysis, collected the data, carried out the literature research and wrote the manuscript. Leticia Altuna Tellechea contributed with the morphological examination and literature research. Marta Alonso Varela performed the flow cytometry analysis. Ignacio Diez de Lezcano Manrique deLara performed de pathological analysis of the iliac lesion

## Funding

None.

## Conflicts of interest

The authors of this paper have no conflicts of interest.
